# Addressing Gaps in Infection Control: Insights from a Nationwide Survey of EMS and Fire Personnel

**DOI:** 10.1017/ash.2025.389

**Published:** 2025-09-24

**Authors:** Leomar White, Arabel Severe, Lorelei Herman, Christine McGuire-Wolfe

**Affiliations:** 1 University of South Florida; 2 USF College of Public Health; 3 University of South Florida College of Public Health; 4 University of South Florida - College of Public Health

## Abstract

**Introduction:** First responders, including EMS and fire personnel, are essential to public safety, often facing high-risk environments with infectious agents. However, IPC training varies across the U.S., creating preparedness gaps. This study identifies training needs through a nationwide survey to offer evidence-based recommendations for effective IPC training, improving safety for first responders and the communities they serve. **Methods:** This study used an online survey to gather feedback from first responders (n=183) across all 50 states. We used the convenience sampling method to select participants based on contact information obtained from the department’s website (n=1,208). We collected demographic data and asked participants to choose from 18 IPC topics they believed should be included in the training, with an option to suggest additional topics. Respondents also rated the factors that influenced training effectiveness and indicated motivations for completing IPC training. An open-ended section allowed participants to share further opinions on IPC for EMS and fire services. **Results:** The most requested infection control topics were decontamination of apparatus and equipment (n=107, 7.21%), use of personal protective equipment (n=104, 7.01%), and transmission and types of communicable diseases (n=99, 6.67% each). The factors rated as most important in determining the effectiveness of training for EMS and fire personnel included “Examples of how the information applies to EMS and fire personnel” (n=76), “Inclusion of scenarios or real-world situations” (n=71), and “Availability of materials in a web-based format” (n=71). The main motivating factors for the completion of infection prevention and control training were identified as “personal health and safety” (n=99, 15.99%), “safety and health of my family” (n=93, 15.02%), and “safety and health of the patients” (n=90, 14.54%). **Conclusion:** These results signal that practical training with examples related to their field is preferred. A greater emphasis on real-life examples and web-based materials shows that the preference goes toward training that is relevant to daily operations and accessible in flexible formats. The first three motivational factors listed above emphasize the personal and professional stakes that first responders have in undergoing this training. This would therefore suggest that infection prevention programs need to be tailored to address the specific risks that first responders face and be delivered in ways that maximize engagement and practical application.

